# Seroprevalence of small ruminant brucellosis in the state of Qatar

**DOI:** 10.1002/vms3.1355

**Published:** 2024-01-23

**Authors:** Hashim Alhussain, Ahmed Gawish, Susu Zughaier, Abdulaziz M. Al‐Zeyara, Hadi M. Yassine, Asmaa Al Thani, Nahla O. Eltai

**Affiliations:** ^1^ Biomedical Research Center Qatar University Doha Qatar; ^2^ Al Maha for Veterinary & Agriculture Services Doha Qatar; ^3^ Faculty of Medicine Qatar University Doha Qatar; ^4^ Department of Animal Resources Ministry of Municipality Doha Qatar

**Keywords:** brucellosis, goat, livestock, Qatar, seroprevalence, sheep, small ruminants

## Abstract

**Background:**

Brucellosis is one of the most important zoonotic diseases worldwide, with a significant economic and health burden. The disease is endemic in many regions around the world. Data regarding the disease in the Arabic Gulf region is scarce, and a limited number of studies have been conducted in Qatar. This study is the first to investigate the seroprevalence of small ruminant brucellosis in the state of Qatar.

**Methods:**

The country was divided into three zones based on animal population (high, medium and low). A total of 494 blood samples from 57 small ruminant flocks were randomly collected from the three zones. Rose Bengal and competitive enzyme‐linked immunosorbent assay were used to investigate the apparent and true seroprevalence at both the animal and flock levels. A regression model was used to investigate the potential risk factors, including geographic zone, sex and age.

**Results:**

At the animal level, the overall (sheep and goat) apparent and true seroprevalence were 9.6% (95% confidence interval [95% CI]: 7.3–12.5) and 8.4% (95% CI: 5.9–11.5), respectively. In sheep, the apparent seroprevalence was 16.7% (95% CI: 12.5–21.8), and the true seroprevalence was 16.1% (95% CI: 11.6–21.7), whereas in goats, the apparent seroprevalence was 2.4% (95% CI: 1.1–5.2), and the true seroprevalence was 0.7% (95% CI: −0.7 to 3.7). At the flock level, overall, apparent sheep and goat seroprevalence were 19.3% (95% CI: 11.1–31.3), 30.0% (95% CI: 16.1–49.8) and 7.4% (95% CI: 2.1–23.4) and true seroprevalence was 18.9% (95% CI: 10.1–31.9), 30.5% (95% CI: 16.1–49.8) and 6.1% (95% CI: 0.3–23.3) respectively. Univariable regression analysis revealed age as the only significant risk factor in the sampled population.

**Conclusions:**

*Brucella* appears to be endemic to small ruminants in Qatar. The findings of this study provide baseline data regarding small ruminant brucellosis in Qatar and will aid in plans to control and eradicate the disease.

## Graphical abstract

- Brucellosis was found to be endemic in small ruminants in Qatar. The disease is more prevalent in sheep (16.7%) compared to goats (2.4%).
- The seroprevalence at the flock level was 19.3% in small ruminates (sheep and goat combined), 30.0% in sheep and 7.4% in goats.
- The disease is more prevalent in Shahaniya and Rayyan municipalities but is present throughout the country among small ruminants. The findings of this study provide baseline data regarding small ruminant brucellosis in Qatar.

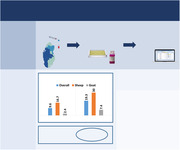

## 1 INTRODUCTION

Brucellosis, also known as undulant, Malta and Mediterranean, is the most common zoonotic disease worldwide (Godfroid, 2017). The disease is caused by *Brucella* species, a small facultative gram‐negative intracellular coccobacilli bacterium (Buttigieg et al., 2018). It was first identified in 1887 by Sir David Bruce in Malta, and later, the disease was named after him in honour of his contribution (Pradeepkiran et al., 2021).

Historically, the genus *Brucella (B)* contained six species. Of the six species, the four pathogenic to humans are *B. abortus*, which is associated with cattle and camels; *B. melitensis* associated with small ruminants and camels; *B. suis* associated with pigs, reindeer and hares; and *B. canis* associated with dogs (Liu et al., 2020). The other two species are *B. ovis*, which infects sheep, and *B. neotomae*, which infects desert woodrats (Rajendhran, 2021). Additional classical and non‐classical species have been added to the genus *Brucella* overtime during the past and current centuries, including *B. pinnipedialis*, *B. microti* and *B. ceti* (Moreno, 2021). Of the four species that can infect humans, the most common sever is *B. melitensis* (García‐Méndez et al., 2019).

In animals, the disease causes abortion, infertility and decreased milk production (Bundle & McGiven, 2017). As antibiotic treatment is not financially feasible, culling is usually the fate of infected animals (Elderbrook et al., 2019). These results are significant economic losses to both the county and animal owners. The annual median loss due to livestock brucellosis in India is estimated at 3.4 billion US dollars (Singh et al., 2015). The disease is transmitted among animals mainly by ingesting contaminated feed and water, sexual contact or direct contact with placenta fetuses or uterine secretion (Geletu et al., 2021; Natesan et al., 2021).

Brucellosis is endemic in most developing countries and is very common in the Mediterranean region (Sarrou et al., 2017). In the Gulf Cooperation Council (GCC) region, including Qatar, the disease is prevalent in animals and humans. The seroprevalence of camel brucellosis in Qatar is relatively high (Alhussain et al., 2022); however, no studies relating to brucellosis on small animals have been conducted. Sheep and goats are the most common livestock animals in Qatar. They constitute the primary meat sources, in addition to their other dairy products. Moreover, sheep sacrificing is associated with several religious rituals and occasions, such as Eid al‐Adha and the naming ceremony of newborns. This study aimed to investigate the seroprevalence of small ruminant brucellosis across the state of Qatar and pave the road for future research in animal brucellosis in Qatar by providing baseline data.

## 2 METHODS

### 2.1 Inclusion criteria

The study included sheep and goats of any age or sex with no history of brucellosis or vaccination and no apparent illness.

### 2.2 Area and study design

The study covered the entirety of the state of Qatar, comprising eight municipalities, namely Doha, Rayyan, Shahaniya, Khor, Daayen, Wakrah, Shamal and Umm‐Salal. According to the Ministry of Municipality (MM) there are 967,253 small ruminants in Qatar, 646,408 are sheep and 320,845 goats (MM, personal communication, 2019). Some municipalities house many small ruminants, while others contain few. Doha municipality has no small ruminants. Based on the animal census in each municipality, we divided the state of Qatar in this study into high (municipalities housing more than 200,000 small ruminants), medium (between 100,000 and 200,000) and low population zones (less than 100,000). The high‐population zone comprises Rayyan and Shahaniya municipalities, the medium‐population zone comprises Khor and Daayen, and the low‐population zone includes the remaining four municipalities (Doha, Wakrah, Shamal and Umm‐Salal). A stratified cross‐sectional study design was used in this study, where each population zone constituted a separate stratum.

### 2.3 Sample size

The minimum required sample size was calculated based on the equation.

$$N=\frac{4z_{\alpha }^{2}p\left(1-p\right)}{W^{2}}={\left(\frac{z_{\alpha }}{E}\right)}^{2}p(1-p),$$

where *N* is the sample size; *p* is the expected proportion who have Brucellosis; *W* is the width of the confidence interval (CI) (equal to twice the margin of error); *E* is the margin of error (half the width, *W*) and *Z_α_* is a value from the normal distribution representing the confidence level, equal to 1.96 at 95% confidence level, (Hulley et al., 1994). Considering the estimated seroprevalence of 20% brucellosis among livestock, and if *W* = 0.1, our minimum required size will be 246 for each animal type. The estimated seroprevalence was based on this research team's previous study on camels (Alhussain et al., 2022).

### 2.4 Animal and flock selection

Animals and flocks were selected randomly. Members of the research team identified the small ruminant flocks housing farms in each population zone. Each animal flock was assigned an identification serial number, which was then used to randomly select flocks from each population zone using SPSS statistics 26 (Statistical Package for the Social Science; SPSS Inc.). From each selected flock, a minimum of 5 and a maximum of 10 samples were collected to increase the number of flocks tested.

All livestock animals in the state of Qatar are tagged with a 15‐digit animal identification number. The animal identification numbers were used to randomly choose animals from the previously selected flocks using SPSS.

### 2.5 Sample collection and preparation

A data collection sheet was used in the field to record the animal type, flock serial number, animal identification number, sex, age and municipality.

Samples were collected over 1 year, starting in November 2019 and ending in November 2020. A licensed veterinarian collected around 5 mL of blood from the jugular vein in serum separator tubes (BD SST II Advance). Collected samples were then transferred inside a cool box (4–8°C) to the Microbiology lab, Biomedical Research Center. In the lab, the blood was centrifuged at 3000 *g* for 10 min to separate the serum and then stored at −20°C in 1.5 mL Eppendorf tubes until the testing time.

### 2.6 Serological tests

Each sample was tested by both Rose Bengal (RBT) (BENGATEST, SYNBIOTICS Europe‐2, RUE Alexander Fleming‐69367 LYON CEDEX 07) and competitive enzyme‐linked immunosorbent assay (C‐ELISA) (SVANOVIR Brucella‐Ab c‐ELISA). In this study, only samples that tested positive by both tests were considered seropositive. Seronegative samples were those that tested negative in one or both tests. A flock was considered seropositive if it constituted one seropositive animal.

RBT was performed following the manufacturer's instructions, as explained in a previous study (Alhussain et al., 2022). Samples were considered RBT positive if agglutination was observed after 4 min of agitation. As for C‐ELISA, the assay was performed per the kit manufacturer's instruction (Alhussain et al., 2022). The per cent inhibition value was calculated to determine the positivity of the samples as follows: $100-({\mathrm{sample}}^{\prime }s\;\mathrm{optical}\;\mathrm{density}\;\times 100\;÷\;\mathrm{conjugate}\;{\mathrm{control}}^{\prime }s\;\mathrm{optical}\;\mathrm{density})$. Samples with a 30% and above percent inhibition value were considered C‐ELISA positive.

### 2.7 Statistical analysis

Data collected from the field were transferred to SPSS (Rogan & Gladen, 1978), and formula was used to estimate the true seroprevalence (TSp) at animal and flock levels. A sensitivity of 94.3% and specificity of 98.2% were adopted from the Minas et al. (2007) study. Wilson score intervals were used to calculate the 95% CI for the apparent seroprevalence (ASp). In comparison, Blaker's interval was used to calculate the 95% CIs for TSp following the recommendation of EpiTools epidemiological calculator's authors (Sergeant, 2018). Clopper–Pearson exact was used for all other statistics to calculate the 95% CIs. Univariable regression tests using SPSS were performed to evaluate sex, age and geographic location as risk factors. The terminology was used to describe regression model types in this article was based on paediatric and perinatal epidemiology journal recommendations (Peters, 2008). A probability value (*p*‐value) of less than 0.05 was considered statistically significant.

## 3 RESULTS

A total of 494 blood samples were collected from 57 small ruminant flocks. Of these, 364 (74%) were females, whereas 130 were males. Of the 494 samples, 247 were sheep (159 females and 88 males) from 30 flocks and 247 goats (205 females and 42 males) from 27 herds. The number of samples collected from each zone was proportional to the animal type census in each location, as shown in Table 1.

**TABLE 1:** Animal census and number of samples collected for each population zone.

Zone	Small ruminants (sheep and goat)	Sheep	Goat
High‐population			
Animal census	487,489 (50.4%)	313,753 (48.5%)	173,736 (54.1%)
No. of samples	254 (51.3%)	120 (48.5%)	134 (54.1%)
Medium‐population			
Animal census	269,859 (27.9%)	196,143 (30.3%)	73,716 (23.0%)
No. of samples	132 (26.7%)	75 (30.3%)	57 (22.9%)
Low‐population			
Animal census	209,905 (21.7%)	136,512 (21.1%)	73,393 (22.9%)
No. of samples	109 (22.0%)	52 (21.1%)	57 (22.9%)

During sample collection, animals were divided into four categories according to age: lambs (less than 1‐year‐old), yearlings (1–2 years old), adults (3–5 years old) and aged (6 years and older).

### 3.1 Seroprevalence at the animal level

Of the 494 samples, 10.0% (95% CI: 7.6–21.9) were RBT seropositive, and 14.0% (95% CI: 11.2–17.4) were C‐ELISA seropositive. In sheep, 17.5% (95% CI: 13.2–22.7) and 24.0% (95% CI: 19.1–29.7) were RBT and C‐ELISA seropositive, respectively, whereas 2.4% (95% CI: 1.1–5.2) and 4.1% (95% CI: 2.2–7.3) were RBT and C‐ELISA seropositive in goats, respectively. Table 2 displays the ASp and TSp at the animal level.

**TABLE 2:** Apparent and true seroprevalence (TSp) at the animal level.

	Seroprevalence	95% CI
Small ruminant		
ASp	9.6% (47/494)	7.3–12.5
TSp	8.4%	5.9–11.5
Sheep		
ASp	16.7% (41/247)	12.5–21.8
TSp	16.1%	11.6–21.7
Goat		
ASp	2.4% (6/247)	1.1–5.2
TSp	0.7%	−0.7 to 3.7

Abbreviations: Asp, apparent seroprevalence; 95% CI, 95% confidence interval.

**FIGURE 1:**
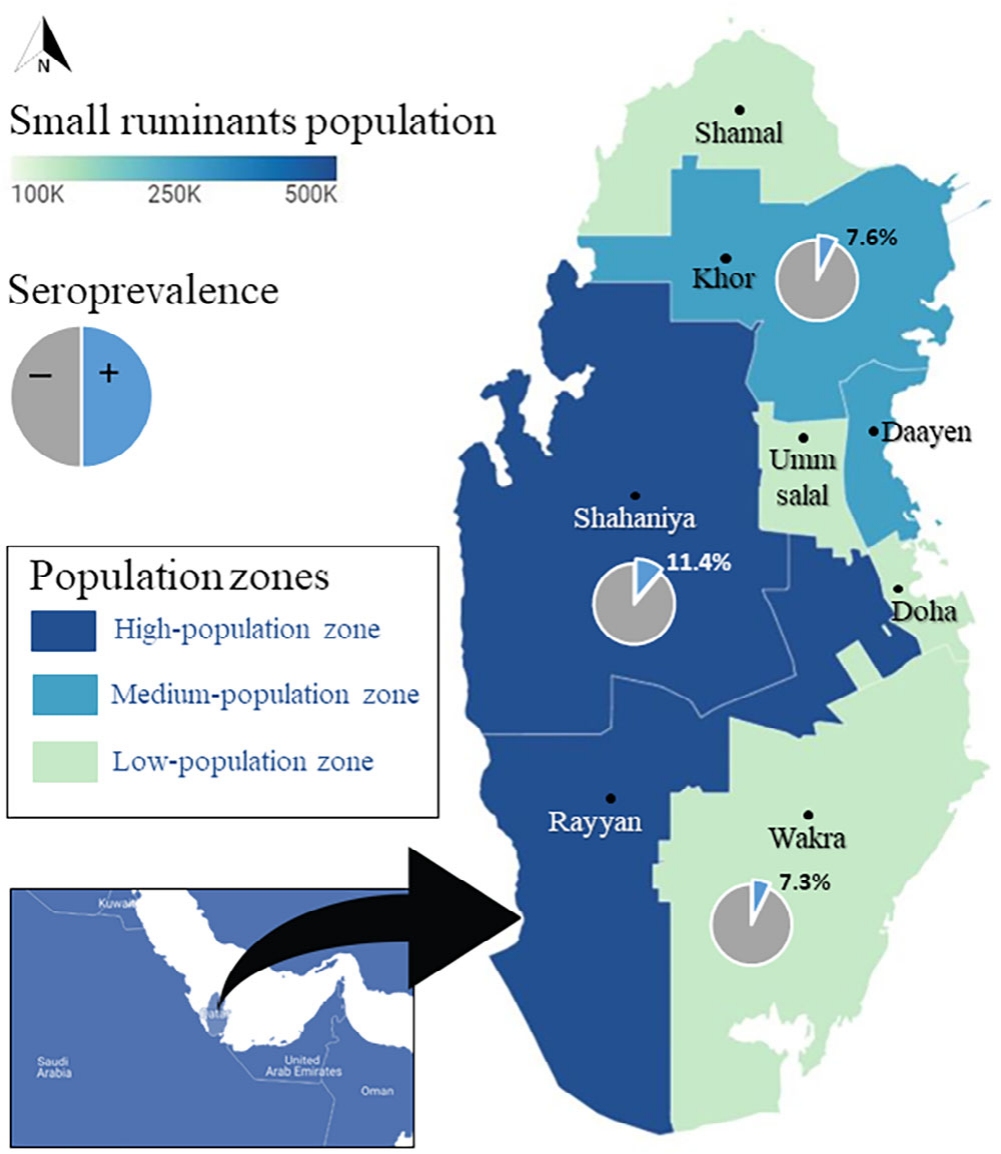
Choropleth map of the state of Qatar displaying small ruminant population distribution, the three population zones and seroprevalence at the zone levels.

The population zone with the highest seropositivity was the high‐population zone, with a seropositivity of 11.4% (95% CI: 7.8–16.0). In comparison, medium and low‐population zones had very similar seropositivity levels of 7.6% (95% CI: 3.7–13.5) and 7.3% (95% CI: 3.2–14.0), respectively (Figure 1).

In sheep, the seropositivity was highest in the high‐population zone with a seropositivity of 19.2% (95% CI: 12.6–27.4), followed by the low‐population zone 15.4% (95% CI: 6.9–25.1), and the lowest seropositivity was in the medium‐population zone with a seropositivity of 13.3% (95% CI: 6.6–23.2). In goats, only six samples were seropositive, all from the high‐population zone.

Overall, 74.0% of the animals were female. In sheep, 64.6% (159/247) were females, and 35.4% were males, whereas in goats, 83.3% (205/247) were females and 16.7% males. The seropositivity was higher in females. In sheep, 18.2% (95% CI: 12.1–25.0) (29/159) were seropositive, whereas 13.8% (95% CI: 7.3–22.9) (12/87) of males tested seropositive. All six goat‐positive samples were females.

Animals were categorized based on their age, as aforementioned. Only two animals were lambs; both were seronegative sheep. None of the aged animals were seropositive; hence, to facilitate further analysis, the animals were regrouped into two main age categories: young (lambs and yearlings) and old (adult and aged).

Two hundred and seventy‐one (55.1%) of the 494 animals were young, and 221 (44.9%) were old. In sheep, 47.6% and 52.4% were young and old, respectively, whereas 62.6% of goats were young and 37.4% were old. In sheep, the seroprevalence in young animals was 22.2% (95% CI: 15.1–30.8) and 11.6% (95% CI: 6.7–15.5) in old sheep. The six seropositive goat samples were all young.

### 3.2 Seroprevalence at the flock level

Eleven out of the 57 flocks were seropositive, 9 were sheep, and 2 were goats. The ASp and TSp are shown in Table 3.

**TABLE 3:** Apparent and true seroprevalence (TSp) at the flock level.

	Seroprevalence	95% CI
Small ruminant		
Asp	19.3% (11/57)	11.1–31.3
TSp	18.9%	10.1–31.9
Sheep		
Asp	30.0% (9/30)	16.7–47.9
TSp	30.5%	16.1–49.8
Goat		
Asp	7.4% (2/27)	2.1–23.4
TSp	6.1%	0.3–23.3

Abbreviations: Asp, apparent seroprevalence; 95% CI, 95% confidence interval.

### 3.3 Risk factor analysis

All the risk factor analysis was conducted on sheep as the number of goat seropositive samples was low. Because all six seropositive goat samples were from the same categories, all were young female animals from the high‐population zone. Univariable regression analysis was done to assess the risk of each factor separately (i.e., population zone, sex and age). The data of the univariable analysis are shown in Table 4.

**TABLE 4:** Univariable analysis of population‐zone, sex and age as risk factors for sheep brucellosis.

	Seroprevalence	Odd ratio (95% CI)	*p*‐Value
Population‐zone			
High	19.2% (23/120)	1.3 (0.6–3.2)	0.54
Medium	13.3% (10/75)	0.9 (0.3–2.3)	0.75
Low	15.4% (8/52)	Ref.	
Sex			
Male	13.8% (12/87)	0.7 (0.4–1.5)	0.37
Female	18.2% (29/159)	Ref.	
Age			
Young	22.2% (26/117)	2.2 (1.1–4.3)	0.03
Old	11.6% (15/129)	Ref.	

Abbreviation: 95% CI, 95% confidence interval.

Multivariable analysis was not needed as the only significant factor was age, and the *p*‐values of the population zone and sex were all >0.1. The univariable analysis showed that young sheep were more than twice as likely to be seropositive.

## 4 DISCUSSION

This is the first study investigating small ruminant brucellosis in the state of Qatar. Human brucellosis is prevalent in Qatar (Rahil et al., 2014) and endemic in camels (Alhussain et al., 2022), but no studies on bovine brucellosis have been conducted. The seroprevalence of camel brucellosis is estimated to be between 15.7% and 26.1%, which is alarming and calls for urgent intervention (Alhussain et al., 2022). Small ruminants in the current study had a seroprevalence of 9.6% (95% CI: 7.3–12.5). This suggests that the disease might be endemic among different types of livestock in Qatar.

Interestingly, the seroprevalence was significantly lower in goats, with an ASp of only 2.4% (95% CI: 1.1–5.2) compared to 16.7% (95% CI: 12.5–21.8) in sheep. Although many studies show a higher seroprevalence of brucellosis in sheep compared to goats, the difference between the two is usually not as wide as in the current study (Boukary et al., 2013; Geletu et al., 2021). The tendency of sheep to congregate at night and in parturition, a behaviour not displayed in goats, may explain, to a certain degree, the disparity in seroprevalence (Jabary & Al‐Samarraee, 2015). Another hypothesis is that the seroprevalence of sheep and goats in Qatar reflects and is affected by the state of brucellosis in the different countries from which a large proportion of these animals are imported.

As for the prevalence of small ruminant brucellosis in the GCC countries, the data are scant. The few available studies usually reported a low prevalence. A seroprevalence of 0.5% for sheep and goats was reported in a study investigating the seroprevalence of brucellosis among sheep and goats across various parts of the Arabic Gulf region (Ebid et al., 2020). Another survey from Oman reported a 0.4% seroprevalence in sheep and goats (Al‐Rawahi, 2015). However, a recent large‐scale study from Kuwait reported a higher seroprevalence of 7% in sheep in districts devoid of vaccination and 4.7% in herds with a vaccination history (Al‐Sherida et al., 2020). In general, brucellosis appears endemic in some, if not all GCC countries with varying prevalence.

The distribution of small ruminants is not even across the state of Qatar. Half the goats and sheep are in Shahaniya and Rayyan municipalities (high‐population zone), about a third is in Khor and Daayen (medium‐population zone), and the remainder is distributed among the remaining municipalities (low‐population zone). Overall, the seroprevalence was higher in the high‐population zone and lower in the low‐population zone. In sheep, the low‐population zone showed a higher seroprevalence, 15.4% (95% CI: 6.9–25.1), than the medium‐population zone, 13.3% (95% CI: 6.6–23.2). However, no statistically significant difference in seroprevalence was identified between the three zones, both overall and among sheep. This is probably due to the small country's size with similarities in geography, weather and animal‐rearing habits. Furthermore, the practice of exchanging and trading animals between livestock owners throughout the country without testing the animals might be a factor.

The seroprevalence was higher in females. All seropositive samples from goats were females, and 70.7% of all seropositive samples from sheep were females. Female sheep had a seroprevalence of 18.2% (95% CI: 12.1–25.0) compared to 13.8% (95% CI: 7.3–22.9) in males. Although the seroprevalence was higher in females, this was not statistically significant. Various studies demonstrated conflicting results regarding sex as a risk factor. Although some studies reported statistically significant higher seroprevalence in females (Teklue et al., 2013), others reported higher seroprevalence in males (Gabli et al., 2015). Numerous studies reported no statistically significant difference in seroprevalence between males and females (Ebid et al., 2020; Elderbrook et al., 2019; Geletu et al., 2021). We think pregnancy, not sex, might be a risk factor. This is supported by a study investigating cattle brucellosis seroprevalence; in that study, higher parity was a significant risk factor (Ndazigaruye et al., 2018). Additionally, studies demonstrating higher female seroprevalence usually ascribe it to the increased erythritol production in placental trophoblast during the later stage of pregnancy (Teklue et al., 2013).

Young animals were more likely to be seropositive. All seropositive goat samples were of young animals, and the seroprevalence in young sheep was much higher than in old sheep, with an odd ratio of 2.2 (95% CI: 1.1–4.3). This finding contrasts other studies and begs for an explanation as other studies consistently reported higher prevalence in older animals regardless of the animal type (Alrawahi et al., 2019; Elderbrook et al., 2019; Geletu et al., 2021; Saeed et al., 2020). It is unclear why younger animals had a higher seroprevalence in this study; however, one possibility could be the sample size, which might not be sufficient to investigate individual risk factors accurately. Another possibility might be the presence of antibodies in brucellosis; recently infected animals will develop IgM. After that, they will produce IgG, then in the chronic form, IgA antibodies, which are more localized; perhaps that might explain the higher seropositivity in younger animals as older animals could be more chronically affected.

At the herd level, the seroprevalence was 19.3% (95% CI: 11.1–31.3), 30% (95% CI: 16.7–47.9) and 7.4% (95% CI: 2.1–23.4) in overall, sheep and goats, respectively. The seroprevalence was much higher at the herd than at the animal level. This is a common and consistent finding (Elderbrook et al., 2019). In a study investigating camel brucellosis in Qatar, the seroprevalence was 20.6% at the animal level and 60.7% at the herd level (Alhussain et al., 2022). This shows that although few animals are infected within the flock, many flocks contain at least one infected animal. Factors that might explain the elevated flock seroprevalence include purchasing animals from livestock fairs and the introduction of purchased animals into the flock without testing for brucellosis (Ebid et al., 2020; Natesan et al., 2021). In addition, the habit of exchanging animals between farm owners for breeding and other purposes in the state of Qatar might be a contributing factor (Alhussain et al., 2022).

In summary, brucellosis appears to be endemic to small ruminants in Qatar. The seroprevalence is high in sheep and is interestingly low in goats. The seroprevalence increases in municipalities with higher animal populations but without statistical significance. The disease affected more females; oddly, the seroprevalence was higher in younger animals. The need to control this disease in Qatar is evident. Measures including vaccination programmes, increasing public awareness and encouraging good biosecurity practices on animal farms are recommended. Further studies are needed to strengthen this study's results and investigate the prevalent species and the status of *B. ovis* in sheep in Qatar. In addition, risk factors associated with small ruminant brucellosis in Qatar need further investigation.

## AUTHOR CONTRIBUTIONS

*Data curation; formal analysis; methodology; visualization; writing – original draft preparation*: Hashim Alhussain. *Methodology; writing – review and editing*: Ahmed Gawish. *Supervision; writing – review and editing*: Susu Zughaier. *Writing – review and editing*: Abdulaziz M. Al‐Zeyara. *Validation; writing – review and editing*: Hadi M. Yassine. *Project administration; writing – review and editing*: Asmaa Al Thani. *Conceptualization; data curation; funding acquisition; investigation; methodology; project administration; resources; supervision; validation; visualization; writing – review and editing*: Nahla O. Eltai.

## CONFLICT OF INTEREST STATEMENT

The authors have no conflicts of interest to declare that are relevant to the content of this article.

## ETHICS STATEMENT

The ethics governing the use and conduct of experiments on animals were strictly observed, and Qatar University's Institutional Biohazard Committee approved the experimental protocol under approval number QU (QU‐IBC‐2019/060‐REN1).

### PEER REVIEW

The peer review history for this article is available at https://publons.com/publon/10.1002/vms3.1355.

## Data Availability

None.
